# Personal Exposure to Household Particulate Matter, Household Activities and Heart Rate Variability among Housewives

**DOI:** 10.1371/journal.pone.0089969

**Published:** 2014-03-03

**Authors:** Ya-Li Huang, Hua-Wei Chen, Bor-Cheng Han, Chien-Wei Liu, Hsiao-Chi Chuang, Lian-Yu Lin, Kai-Jen Chuang

**Affiliations:** 1 Department of Public Health, School of Medicine, College of Medicine, Taipei Medical University, Taipei, Taiwan; 2 School of Public Health, College of Public Health and Nutrition, Taipei Medical University, Taipei, Taiwan; 3 Department of Cosmetic Application and Management, St. Mary's Junior College of Medicine, Nursing and Management, Yilan, Taiwan; 4 Department of Information Management, St. Mary's Junior College of Medicine, Nursing and Management, Yilan, Taiwan; 5 Division of Pulmonary Medicine, Department of Internal Medicine, Shuang Ho Hospital, Taipei Medical University, Taipei, Taiwan; 6 School of Respiratory Therapy, College of Medicine, Taipei Medical University, Taipei, Taiwan; 7 Department of Internal Medicine, Division of Cardiology, National Taiwan University Hospital, Taipei, Taiwan; Medical University Innsbruck, Austria

## Abstract

**Background:**

The association between indoor air pollution and heart rate variability (HRV) has been well-documented. Little is known about effects of household activities on indoor air quality and HRV alteration. To investigate changes in HRV associated with changes in personal exposure to household particulate matter (PM) and household activities.

**Methods:**

We performed 24-h continuous monitoring of electrocardiography and measured household PM exposure among 50 housewives. The outcome variables were log_10_-transformed standard deviation of normal-to-normal (NN) intervals (SDNN) and the square root of the mean of the sum of the squares of differences between adjacent NN intervals (r-MSSD). Household PM was measured as the mass concentration of PM with an aerodynamic diameter <2.5 µm (PM_2.5_). We used mixed-effects models to examine the association between household PM_2.5_ exposure and log_10_-transformed HRV indices.

**Results:**

After controlling for potential confounders, an interquartile range change in household PM_2.5_ with 1- to 4-h mean was associated with 1.25–4.31% decreases in SDNN and 0.12–3.71% decreases in r-MSSD. Stir-frying, cleaning with detergent and burning incense may increase household PM_2.5_ concentrations and modify the effects of household PM_2.5_ on HRV indices among housewives.

**Conclusions:**

Indoor PM_2.5_ exposures were associated with decreased SDNN and r-MSSD among housewives, especially during stir-frying, cleaning with detergent and burning incense.

## Introduction

Air pollution exposure, particularly particulate matter (PM), has been associated with increased cardiovascular mortality and morbidity [1], [2]. These associations have been partially supported by the association of PM with heart rate variability (HRV) changes, and previous panel studies have reported this association as a possible mechanism linking PM to increased risk for cardiovascular diseases [3]. Recently, several studies have reported that the association of cardiovascular endpoints with personal exposure to PM [4]–[6]. It is also known that people spend 87% of their time in enclosed buildings [7]. These findings imply that exposure to indoor PM may increase the cardiovascular effects of PM exposure. Moreover, the World Health Organization (WHO) considers indoor air pollution as the 3^rd^ most important risk factor, responsible for 4.3% of the global burden of disease [8].

There are many household sources of PM, such as cooking, cleaning and tobacco smoke [7], [9], [10], which worsen indoor air quality and induce cardiovascular effects among housekeepers who spend most of their time in houses. However, studies of the adverse effect on HRV of personal exposure to household PM among housekeepers are lacking. The effect modification of household activities on the association of PM exposure with HRV changes remains unclear. The aim of this study was to investigate the association between personal exposure to household PM and HRV indices among housewives using four 24-h visits to monitor each participant's household PM exposure, HRV indices and household activity pattern in their private homes.

## Materials and Methods

### Ethics approval

The study was approved by the ethics committee of St. Mary's Medicine Nursing and Management College. Written informed consent was obtained from each participant before the study began.

### Study participants and design

This panel study was designed to simultaneously and continuously monitor changes in household PM levels and HRV indices as well as household activities among housewives in their own homes. The selection criteria for volunteer participants were as follows: no history of smoking or drinking; no medication that might affect cardiac rhythm; and no cardiovascular diseases, such as coronary artery disease, arrhythmia, hypertension, diabetes mellitus and dyslipidemia. Ninety-two housewives responded to our recurring advertisement; 50 of them (54%) living in 50 homes in the Taipei metropolitan area met the criteria and were willing to participate in this study after our protocols had been explained.

The protocol included four home visits that entailed continuous 24-h monitoring of electrocardiography (ECG), household PM, noise, meteorological conditions and time-activity patterns at approximately one-season intervals in the years 2010 to 2012. Each of the 50 housewives had four home visits, for a total of 200 home visits. During their first visits, age, sex, and household characteristics were recorded using a questionnaire. Height and weight were measured and used to calculate body mass index (BMI). Information on indoor environmental measurements and time-activity patterns during the study periods were collected at all visits.

### Household particulate matter, meteorological conditions, noise and time-activity patterns

We conducted 24-h continuous monitoring of household PM, meteorological conditions, noise and time-activity patterns during each visit for each housewife. Household PM less than 2.5 µm in diameter (PM_2.5_), temperature and relative humidity were measured continuously using a personal dust monitor (DUSTcheck Portable Dust Monitor, model 1.108; temperature and humidity sensor, model 1.153FH; Grimm Labortechnik Ltd., Ainring, Germany), which measured and recorded 1-min mass concentrations of PM_2.5_ as well as temperature and relative humidity. Noise level was measured using a portable noise dosimeter (Logging Noise Dose Meter Type 4443, Brüel & Kjær, Nærum, Denmark), which reported 1-min continuous equivalent sound levels (Leq) and the time-weighted-averages (TWAs) of noise doses. A range of 30–100 dBA was used to measure noise exposure with 1-min readings over 24 hours.

We asked each participant to carry the dust monitor personally from 0700 hr to 2200 hr to measure personal household PM_2.5_ and noise exposure during the participants' household activities. Participants themselves recorded their time-activity patterns, such as indoor tobacco smoke exposure, indoor chemical dispersion, burning incense, stir-frying and cleaning with detergent every hour from 0700 hr to 2200 hr during the study periods. After sampling, the raw data for 1-min household PM_2.5_, temperature, relative humidity and noise measurements were matched with the sampling time of HRV monitoring and then calculated as 1-, 2-, 3- and 4-h means if 75% of the data were present.

### Heart rate variability monitoring

We performed continuous ambulatory ECG monitoring using a PACERCORDER 3-channel device (model 461A; Del Mar Medical Systems LLC, Irvine, CA, USA) with a sampling rate of 250 Hz (4 msec) from 0700 hr to 2200 hr (15 hours) during the study periods. ECG tapes were analyzed using a Delmar Avionics model Strata Scan 563 (Irvine, CA). A complete 5-min segment of the normal-to-normal (NN) interval was taken for HRV analysis, including the standard deviation of NN intervals (SDNN) and the square root of the mean of the sum of the squares of differences between adjacent NN intervals (r-MSSD). Each participant obtained approximately 720 successful 5-min HRV measurements during the four visits (12 measurements for each hour, 180 measurements for each visit) for data analysis. We obtained approximately 32,432 5-min measurements of HRV indices for 50 participants in our data analysis (missing data rate = 9.9%).

### Statistical analysis

Mixed-effects models were used to examine the association between household PM_2.5_ and log_10_-transformed HRV indices by running R statistical software version 2.15.1. The independent variables were the 1-, 2-, 3- and 4-h mean household PM_2.5_, whereas the dependent variables were SDNN and r-MSSD. We treated participant age, BMI, hour of day, temperature, relative humidity, noise, household PM_2.5_ and household activity periods (yes vs. no) including indoor tobacco smoke exposure, indoor chemical dispersion, burning incense, stir-frying and cleaning with detergent as fixed effects and fitted the participant identity number as a random intercept term in our mixed-effects models.

Effect modification by indoor tobacco smoke exposure, burning incense, stir-frying and cleaning with detergent were assessed in separate mixed-effects models by including interaction terms between household PM_2.5_ effects and each potential effect modifier. Household PM_2.5_ effects are expressed as percent changes by interquartile range (IQR) changes as [10(β×IQR)-1]×100% for log_10_-transformed HRV indices, where β is the estimated regression coefficient. Power analysis and sample sizes calculation were performed with power analysis and sample size (PASS) (NSCC, Kaysville, UT, USA). A significance level of 0.05 was used to determine statistical significance in our models.

## Results

Thirty-six thousand 5-min measurements of indoor environmental variables and 32,423 5-min measurements of HRV indices were included in the data analyses. As shown in Table 1, the age range of the 50 housewives was 25–64 years. The mean BMI was 23.2 kg/m^2^. Of the 50 participants, 44 with 157 home visits cooked by stir-frying, 38 participants with 129 home visits cleaned with detergent, and 24 participants with 96 home visits burned incense. Only 2 participants with 6 home visits went out shopping, and 50 participants with 194 home visits stayed home during the study periods. All participants used gas for cooking during the study period.

**Table 1:** Basic characteristics of the 50 participants (Mean ± SD).

Variables	
Age, years	
Mean	38.0±10.5
Range	25–64
Body mass index, kg/m^2^	
Mean	23.2±2.5
Range	19.0–31.0
Household activities among the 50 participants, no (%)	
Indoor tobacco smoke exposure	7 (14)
Incense burning	24 (48)
Cooking with stir-fry	44 (88)
Cleaning with detergent	38 (76)
Indoor chemical dispersion	5 (10)
Shopping	2 (4)
Household activities during 200 home visits, no (%)	
Indoor tobacco smoke exposure	14 (7)
Incense burning	96 (48)
Cooking with stir-fry	157 (78.5)
Cleaning with detergent	129 (64.5)
Chemical indoor dispersion	8 (4)
Shopping	6 (3)

The participants' household PM_2.5_ exposure, meteorological conditions, noise exposure and HRV indices are summarized in Table 2. When the participants' HRV indices were measured during the study periods of the years 2010 to 2012, they demonstrated relatively normal PM levels (WHO air quality guidelines for 24-h mean PM_2.5_: 25 µg/m^3^) [11], with a household PM_2.5_ of 23.5 µg/m^3^ (SD = 19.4 µg/m^3^). The mean noise level was under 50 dBA, which may not enhance sympathetic activity [12]. The mean values (SD) of the log_10_-transformed HRV indices were 1.62 msec (0.32) for SDNN and 1.11 msec (0.28) for r-MSSD.

**Table 2:** Summary statistics of 5-min household PM_2.5_, meteorological conditions, noise and HRV indices for the 50 participants experienced during 200 visits.

Variables	No.	Mean ± SD	Range
Household PM_2.5_, µg/m^3^	36,000	23.5±19.4	12.0–121.0
Noise, dBA	36,000	47.5±22.5	26.0–78.0
Temperature, °C	36,000	24.7±3.6	14.0–33.2
Relative humidity, %	36,000	69.5±3.0	65.1–78.2
Log_10_ SDNN, msec	32,423	1.62±0.32	1.01–2.00
Log_10_ r-MSSD, msec	32,423	1.11±0.28	0.55–1.65

The associations between household PM_2.5_ and log_10_-transformed HRV indices estimated by the mixed-effects models are shown in Table 3. After adjusting the models for age, BMI, hour of day, temperature, relative humidity, noise and household activity periods including indoor tobacco smoke exposure, indoor chemical dispersion, burning incense, stir-frying and cleaning with detergent, household PM_2.5_ exposures significantly decreased SDNN and r-MSSD. Interquartile increases in the 1-, 2-, 3- and 4-h mean household PM_2.5_ (19.8, 17.4, 16.5, and 16.2 µg/m^3^, respectively) were associated with 1.25–4.31% decreases in SDNN. The 2-, 3- and 4- means were associated with 1.96–3.71% decreases in the r-MSSD. The greatest decreases in log_10_-transformed HRV indices occurred at the 4-h mean. Age, BMI, temperature and household activity periods including indoor tobacco smoke exposure, burning incense, stir-frying and cleaning with detergent were significantly associated with decreased SDNN and r-MSSD. No association was observed between relative humidity, noise, indoor chemical dispersion and decreased HRV indices.

**Table 3:** Percentage changes (95% CI)^a^ in HRV indices for interquartile range changes in household PM_2.5_.

Outcome	1-hr mean	2-hr mean	3-hr mean	4-hr mean
Log_10_ SDNN	−1.25	−2.15	−3.02	−4.31
	(−2.00, −0.50)	(−2.77, −1.53)	(−3.87, −2.17)	(−6.50, −2.12)
Log_10_ r-MSSD	−0.12	−1.96	−2.64	−3.71
	(−2.78, 2.54)	(−3.01, −0.91)	(−4.80, −0.48)	(−5.11, −2.30)

a Coefficients are expressed as % changes for interquartile range changes in household PM_2.5_ exposure in models adjusting for age, BMI, hour of day, temperature, relative humidity, noise and household activity periods including indoor tobacco smoke exposure, indoor chemical dispersion, burning incense, stir-frying and cleaning with detergent.

We found a consistent effect modification for household PM_2.5_ by different household activity periods, including stir-frying, cleaning with detergent and burning incense (Table 4). Participants showed changes of −4.52% and −2.94% in SDNN and r-MSSD, respectively, associated with increased household PM_2.5_ during stir-frying, whereas participants showed no significant change in HRV indices during study periods without cooking. We also found relatively stronger effects of household PM_2.5_ on participants during cleaning with detergent compared to study periods without cleaning. A similar result was observed in a model including an interaction term between burning incense and household PM_2.5_, although the statistical significance of the interaction was weaker than those in models evaluating effect modifications by cooking and cleaning. However, no significant interaction was found between indoor tobacco smoke exposure and household PM_2.5_ for HRV indices.

**Table 4:** Effect modification^a^ of the association of HRV indices with interquartile range increases in 4-h mean household PM_2.5_ by household activities.

	Log_10_ SDNN (95% CI)	Log10 r-MSSD (95% CI)
Indoor tobacco smoke exposure		
Yes	−0.99 (−2.22, 0.24)	−1.49 (−2.84, −0.14)
No	0.31 (−1.50, 2.12)	1.52 (−3.07, 6.11)
P-value, interaction^b^	0.458	0.227
Burning incense		
Yes	−2.25 (−4.02, −0.48)	−1.99 (−3.42, −0.56)
No	1.89 (−0.45, 4.23)	0.65 (−1.00, 2.30)
P-value interaction	0.025	0.087
Stir-frying		
Yes	−4.52 (−5.37, −3.67)	−2.94 (−3.86, −2.02)
No	−1.15 (−3.08, 0.78)	0.42 (−1.58, 2.42)
P-value interaction	<0.001	<0.001
Cleaning with detergent		
Yes	−3.38 (−4.78, −1.98)	−2.44 (−4.31, −0.57)
No	−0.68 (−1.25, −0.11)	2.68 (1.57, 3.79)
P-value interaction	0.012	<0.001

a Coefficients are expressed as % changes for interquartile range changes in household PM_2.5_ exposure in models adjusting for age, BMI, hour of day, temperature, relative humidity, noise and interaction terms.

b P-value is for effect modification.

## Discussion

To our knowledge, this is the first study to evaluate the impact of personal exposure to household PM_2.5_ and the impact of household activities on acute changes in HRV indices among housewives. In general, our findings suggest that personal exposure to household PM_2.5_ may impair autonomic function and result in decreased HRV indices. Few studies have investigated the relationship between personal PM_2.5_ exposure and HRV indices [5], [13], [14], and the majority of those have examined effects of ambient PM_2.5_ exposure on autonomic function [15]–[17] in human subjects. Our PM_2.5_-induced HRV reductions are in agreement with previous findings [13]–[17]. The findings support the statement of the American Heart Association's expert panel regarding the biological mechanisms of the effects of PM_2.5_ on cardiovascular events, which are thought to occur through a neural mechanism, altering central nervous system functions [3].

We found that stir-frying and burning incense increased indoor PM_2.5_ levels and that the increase may modify the association between household PM_2.5_ and HRV indices. Epidemiological studies have reported that individuals exposed to indoor cooking oil fumes have a high risk of respiratory diseases [18] and lung cancer [19]. Few panel studies have reported the association between air pollution due to cooking and cardiopulmonary endpoints. In a panel of 387 nonsmoking Chinese restaurant workers, exposure to cooking oil fumes in kitchens was associated with increased urinary 8-OHdG levels. Female workers had a greater oxidative stress response to cooking oil fumes than male workers [20]. Another panel study observed the association between household wood smoke exposure and ST-segment depression in a panel of 70 women using open wood fires for cooking [21]. The present study showed the effect modification of stir-frying on the association between household PM and HRV reduction. Burning incense is a long-standing Asian tradition used to give respect to ancestors. It has been reported that Taiwan households worship twice per day and are exposed to high levels of particulate air pollution [22]. A recent epidemiological study has reported that exposure to incense smoke in the home may increase the risk of lung cancer among smokers [23]. An *in vitro* study showed that exposure of human coronary artery endothelial cells to burning incense particles induced cytokine production and reduced nitric oxide formation [24]. Studies evaluating incense PM-induced autonomic dysfunction are lacking. Our study suggested some caution in the use of incense for housewives due to incense PM-induced decreases in HRV indices. Overall, our findings add to the growing evidence that air pollutants from stir-frying and incense burning can induce autonomic dysfunction in human subjects similar to those from vehicle and industrial emissions [13]–[17]. The public health implication is grave because high levels of exposure to indoor air pollution from stir-frying and burning incense are common in Asian countries.

Another interesting finding in our study was that use of detergents when cleaning appeared to modify the effects of household PM_2.5_ on HRV indices; greater household PM_2.5_ effects on HRV indices were observed when participants cleaned with detergent. A recent cross-sectional study used the indoor air quality checklist published by the Department of Occupational Health and Safety to evaluate the health risk of 102 building occupants in a nonindustrial workplace setting. The results showed that the main factors influencing the high number of complaints regarding indoor air quality included indoor detergent and chemical dispersion. Cleanliness led to high pollutant levels and complaints from occupants due to health risks when working inside [10]. Although a limited understanding of the indoor dispersion of detergents and chemicals can make them the primary source of indoor air pollution, odor-related complaints are an example of the human sense of the existence of indoor chemical pollutants. The present study indicated that cleaning with detergent increased the levels of household PM_2.5_ and modified the association between household PM_2.5_ and HRV reduction. These findings have important implications for the feasibility of reliably investigating the associations between cleanliness, indoor air quality and health effects in large-scale epidemiological and intervention studies of household PM. We recommend further studies to investigate the clinical significance of the association between household particle control and cardiovascular health improvement.

Some possible limitations may confound our findings of HRV reduction by household PM_2.5_, including unavailable data on associations with outcomes and some key physiologic and environmental information. First, we could not adjust for respiration-modulated autonomic activity in our study because we were unable to measure key respiration parameters, such as nasal and mouth airflow, chest wall movement and abdominal movement, during the study periods. Second, medication and comorbidity among older housewives could still confound our findings for household PM_2.5_ effects on HRV reduction even though we used strict criteria to exclude cases with chronic cardiopulmonary diseases and specific medication from our study. Third, other unmeasured indoor air pollutants, such as ozone, carbon monoxide and total volatile organic compounds, may have confounded our findings. Fourth, non-randomized recruitment may result in selection bias and confound the association of HRV with household PM_2.5_. Last, the effects of noise and household activities on HRV require further clarification because the sample size of our study may not be large enough to adjust for their effects completely.

### Conclusions

We believe our findings generally indicate that household PM_2.5_ was associated with autonomic function in housewives. Household activities including stir-frying, burning incense and cleaning with detergent were associated with increased levels of household PM_2.5_ and modify its effects on HRV reduction.
